# Dr. Bree, on the Use of Digitalis in Consumption

**Published:** 1799-11

**Authors:** Robert Bree

**Affiliations:** Birmingham


### *To the Editors of the Medical and Phyſical Journal.*

GENTLEMEN,

IF you are of opinion that the bad effects of a medicine merit as much obſervation in practice as its virtues, and think the following caſes and remarks are deſerving attention, I requeſt you to inſert them in your very uſeful and impartial publication. They are a few out of many caſes of phthiſis in which I had tried the power of fox-glove, but they are the only ones on which I could depend, from my actual obſervation of the progreſs and events.

I am, GENTLEMEN,

Your humble ſervant,

BIRMINGHAM, *Oct.* 12, 1799. ROBERT BREE.

---

CASE I.—Mrs. B. aged 25, of a delicate habit and fair complexion, had long been diſpoſed to phthiſis: the preſent ſtate was preceded by hæmoptoe, which had frequently returned in the laſt ſix months. She has now all the ſymptoms of the laſt ſtage, except diarrhœa; her pulſe between the exacerbations of the hectic fever is never leſs than 110. She came out of the country for my attendance, and I had ſeen her very frequently in the laſt two months. May the 16th, I conſulted with Dr. WITHERING* on the caſe as now ſtated. We preſcribed pul. fol. digital. in pills with extract glycyrrhizæ, and directed two grains to be taken at eleven o'clock in the forenoon, and at five in the afternoon, with a ſaline draught. As her ſtrength was greatly diminiſhed, ſhe was allowed ſome porter and a meat diet. I was to viſit her as regularly as poſſible, to mark the effect of this plan, and particularly its influence on the pulſe. May 17th her pulſe was 102—the 18th, 110—the 19th, 97—20th, 98—21ſt, 96—22d, 92—23d, 87. She had been frequently harraſſed with pains in her bowels, and ſhe has now a tendency to diarrhœa, but without relief of pain. She has a total loſs of appetite, and her weakneſs is increaſed. She has much averſion to medicine, but as the pulſe is retarded, Dr. WITHERING united his perſuaſion to mine, and ſhe promiſed to purſue the pills as before. A mixture with chalk was preſcribed to be taken occaſionally. May 24th, pulſe 88.—She has conſtant anorexia, and

* This excellent phyſician has ſince paid the debt of nature, to the general regret of ſcience, and the particular loſs of his medical brethren in this neighbourhood.

and frequent vertigo – her weaknefs is greater—her cough not lefs frequent—the hectic exacerbations as regular, and her fweats as profufe as ever. May 25.—She can no longer be prevailed upon to take the pills, but fhe has purfued them to this day—her pulfe is 96—fhe has a fenfe of weight and oppreffion under the fternum, which before had preceded a great difcharge of pus. May 26.—She throws up matter in large quantities, apparently from a new vomica. The next day her pulfe rofe to its former range, between 100 and 120—fhe would not confent to take any medicine except pills with opium after this date. In a fortnight fhe was fo far recruited in ftrength as to be able to take a daily airing in a carriage for two or three hours, which practice fhe continued for a fhort time; fhe then returned to her home, and died with calmnefs in September.

Case 2.—Mrs. C. a married lady from the country, aged 35, fent for me to her lodgings in this town, July 20th; I found that fhe had fpit blood in April, after having been long fubject to cough and pain of the fide; fhe had received much medical advice with little relief; fhe had now every confirmed fymptom of ulcerated lungs, but there was confiderable ftrength left, and the body was flefhy—hectic fever was, however, regular in its acceffions—her expectoration was very profufe, particularly in the night. I was convinced that it was purulent, from repeated examination. In the middle of the day, when fhe was leaft feverifh, her pulfe was never below 110. In the night, her difficulty of lying down, and want of reft, diftreffed her greatly. I directed a tincture to be prepared with half an ounce of powdered fox-glove leaves in two ounces of proof fpirit, and two ounces of water, and fhe was to begin with only ten drops, and increafe the dofe every fix hours.

I faw her every day once, and frequently twice, but I never found her pulfe lefs frequent than 100 in a minute. Aug. 2d.—She took nearly 40 drops with almond milk every fix hours; her appetite had been growing worfe every day—it is now fo impaired that fhe is difgufted with all food—fhe pukes very feldom—her ftrength is greatly declined—occafionally fhe has lefs cough, but then her difficulty of breathing is increafed—her fpirits are depreffed, and fhe has hyfteric fits of crying: fhe has loofe ftools frequently, which lower her greatly. I now prefcribed an infufion of myrrh and columbo root, with a more cordial diet. I directed her drops to be ftill taken, as fhe had never yet rejected them from her ftomach. Aug. 5.—Her ftrength and appetite are not improved —fhe has more heat and fever; fhe is alfo affected with vertigo, and occafional casional confusion of mind—the pulse at 108—120. Aug. 6.—She is to omit the farther use of the tincture, as her strength and spirits are rather worse, and the nervous system greatly affected. In the evening of this day she was carried to her bed, perfectly helpless. She soon after requested to be placed in the night chair—her attendant complied with her wish, and she immediately died. The waste of flesh was not found so great as is usual in the end of a consumption; and it might be inferred, that she would have lasted longer if the force of her disease had been her only opponent.

CASE 3.—Mrs. H. between 40 and 50 years of age, has had many children, and been long phthisical—in addition she has frequent discharges of matter from an abscess in the perinæum. July 23.—I directed a tincture of fox-glove, as follows:—℞. Fol. digital. purpur. ʒij. spiritus vini ten. aquæ fontis a. a. ℥i. digere per horas 24. et cola.

She at first took fifteen drops of this tincture twice in the day, and afterwards increased the dose. July 30.—She takes 30 drops in a dose, but there has been no change in the pulse, nor in the accessions of hectic chills and heats—her cough is less frequent, and she rests better in the night: this good symptom has before taken place when the abscess in perinæo discharged, which is now the case. Aug. 8.—She takes 40 drops, without puking; but her pulse is never less than 100—her appetite totally gone—her strength diminished, and her cough as bad as ever. Aug. 14.—She finds no amendment of particular symptoms, and much less feeling of comfort. She removed into the country, taking no medicine, and died a few weeks afterwards.

CASE 4.—WILLIAM HARRISON, aged 23, of a thin habit, and long disposed to symptoms of phthisis, was attacked with hæmoptoe a fortnight since, and spit blood five days. May 31.—His cough is frequent, with expectoration of frothy mucus, but no blood—he has a severe pain in his right side, and is often affected with rigors—he does not complain of succeeding heat or fever—p. 100. He was ordered to take two grains of pulv. fol. digital. every twelve hours, with a saline draught; a blister was to be applied to his side. June 10.—He has less cough, but the pulse is not retarded. I have seen him twice only since the 31st of May, but I have been assured that he has taken the medicines regularly. June 14.—Hæmoptoe returned—p. 120. June 19.—The spitting of blood ceased in two days—he has an acute pain in his left side—sickness and sometimes puking distress him—p. 120. His cough is increased, and the expectoration is more considerable—he has fever, with delirium, every night.

night. I thought myſelf no longer juſtified in purſuing the plan I had ſet out with, as he had taken above ſeventy grains of fox-glove, without any progreſs in removing pneumonia, or even hæmoptyſis.

CASE 5.—JOHN PAIN, aged 36, had been affected with phthiſis and hæmoptoe a year. He has a great expectoration of bloody pus, particularly in the night, with regular acceſſions of hectic fever—p. 120. June 28.—I directed a drachm of pulv. digital. to be made into 60 pills, and one to be taken every eight hours. After taking the whole number, his pulſe was not retarded, nor any bad ſymptom removed.

CASE 6.—GEORGE UNDERHILL, aged 28, affected with hæmoptyſis laſt year, with cough and pain of the ſide—his ſpitting of blood has recurred at intervals ever ſince, and has been conſtant for the laſt ten days—he has chills and heats, and night ſweats—p. 120. July 8.—I directed the tincture of fox-glove to be taken in cold milk and water twice a day. He purſued this till anorexia and weakneſs made him loſe all patience; in the mean time his diſeaſe advanced rapidly to the laſt ſtage, and I believe nothing ſtopped its progreſs, though I did not ſee him after he left off the tincture.

CASE 7.—MARY CLARK, aged 32, had been long in a confirmed phthiſis—ſhe had had much advice before ſhe came to me, without benefit—ſhe has no diarrhœa, but every other bad ſymptom. July 10.—I directed the tincture to be taken twice a day. July 12.—She complains of unuſual pain and flatulence in the ſtomach and bowels—I directed pilul. galban. compoſit. to be taken twice a day with chamomile tea. July 17.—Her pulſe is 100—ſhe complains greatly of general weakneſs, anorexia and flatulence—ſhe is always cold—her cough is as bad as ever, but her hoarſeneſs is leſs. July 19.—She is now affected with heats as well as chills—p. 100; ſhe is to continue the tincture, and to take it with a ſaline draught. July 24.—She cannot force herſelf to take any nutriment but tea, and ſuch weak fluids—her weakneſs is extreme, and ſhe attributes it in great meaſure to want of food—ſhe has looſe ſtools—other phthiſical ſymptoms are not removed or leſſened. July 31.—Nothing more favourable having appeared in the caſe, I ordered other medicines.

CASE 8.—Mrs. MOWSLEY, aged 35, has confirmed phthiſis with every bad ſymptom except diarrhœa—it followed hæmoptyſis ſeven months ſince. Sept. 2.—I directed powder of fox-glove to be taken every every eight hours in dofes of one grain and a half each. She purfued this plan with great punctuality till Sept. 12, but her pulfe has not been retarded, nor one bad fymptom removed. At this date her debility and lofs of appetite were fo great that I directed a myrrh mixture with tincture of opium to be taken as well as the digitalis. After continuing thefe medicines a few days longer, without any good effect, fhe refigned herfelf to her fate, which is certain, if not yet arrived.

